# Assessing the Drivers of Distribution for a Cryptic Species Over a Large and Rugged Landscape: Occupancy Modeling of the Critically Endangered Northern White‐Cheeked Gibbon

**DOI:** 10.1002/ece3.72957

**Published:** 2026-01-22

**Authors:** Jay White, Akchousanh Rasphone, Khamkeo Syxaiyakhamthor, Anong Thorya

**Affiliations:** ^1^ Wildlife Conservation Society Vientiane Lao PDR; ^2^ World Wide Fund for Nature in Laos Vientiane Lao PDR

**Keywords:** bioacoustics, forest cover, gibbons, hunting, Laos, *Nomascus*, occupancy modeling, roads

## Abstract

Hunting and wildlife trade in Lao PDR (also known as Laos) has left species of larger wild fauna existing at low densities, almost exclusively in remote areas of rugged forest, and wary of human presence. This makes surveying of larger fauna in the country difficult as observation of individuals is remarkably rare. Due to the critical nature of faunal conservation in Lao PDR (many globally threatened species facing the immediate risk of extirpation) the difficulty of conducting faunal surveys does not supersede the need to do so. Occupancy estimation and modeling, using repeated sampling of presence/non‐detection of a site, offers a potential solution to this challenge. We conducted presence/non‐detection sampling of 80 sites spread across the potentially suitable habitat for the Critically Endangered northern white‐cheeked gibbon in Nam Et—Phou Louey National Park in the northeast of Lao PDR. We used maximum likelihood models to test the significance of several natural and anthropogenic covariates on the population's detection and occupancy probability to assess the drivers of the population's distribution. Detection was found to be a function of cloud cover, the proportion of bamboo forest, and topographic roughness of the site. Occupancy was found to be a function of human usage of the site, distance from the nearest road, and the uninterruptedness of forest cover. These results suggest that the viability of this species in Nam Et—Phou Louey National Park relies on preventing the expansion of all forms of roads into the park, maintaining continuous forest cover, and mitigating human presence in the habitat.

## Graphical abstract

We conducted presence/non‐detection sampling of the Critically Endangered northern white‐cheeked gibbon in Lao PDR. Occupancy was found to be a function of human usage of the site, distance from the nearest road, and the uninterruptedness of forest cover. These results suggest that the viability of this species in Lao PDR relies on preventing the expansion of roads into protected areas, maintaining continuous forest cover, and mitigating human presence in the habitat.

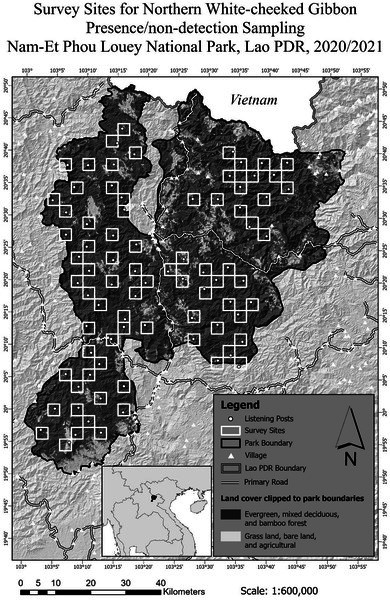

## 1 Introduction

Occupancy estimation and modeling have grown in popularity as a method to survey hard‐to‐detect species and populations existing at low density. In occupancy estimation and modeling, researchers can forgo making density estimates (which can be spurious anyway) by using indices of relative rates of occupancy or use by the population or populations in question to reveal trends in distribution and potential drivers of that distribution (Neilson et al. 2013; MacKenzie et al. 2018; Regan et al. 2018). Due to the high rates of hunting in Lao People's Democratic Republic (hereon, Lao PDR), larger faunal species in the country exist at low densities in difficult‐to‐access areas of remote forest and tend to be wary of human presence, creating significant challenges to conducting surveys of these populations (Duckworth et al. 1999; Gray et al. 2017). This has made occupancy estimation and modeling particularly relevant for wildlife surveys in this region of mainland Southeast Asia (Gray et al. 2010; Hallam et al. 2016; Rasphone et al. 2019, 2021; Vu et al. 2020).

**FIGURE 1:**
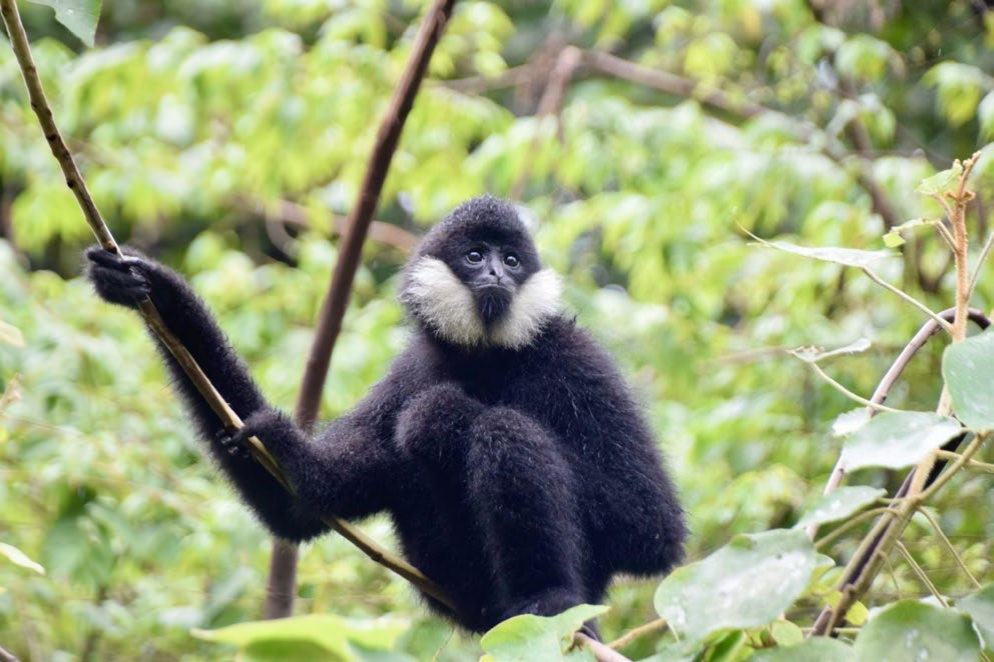
Male northern white‐cheeked gibbon ( *Nomascus leucogenys* ). Photo by Jeremy Phan, Lao Conservation Trust for Wildlife.

Due, in large part, to hunting and habitat loss, gibbons (Hylobatidae) are one of the world's most threatened groups of primates (Duckworth 2008; Rawson et al. 2011; Melfi 2012). Within the family, the rarest genus is *Nomascus* (the crested gibbons) (Mootnick and Fan 2011). Recently extirpated from China (Fan et al. 2013), *Nomascus leucogenys* (northern white‐cheeked gibbons—Figure 1) only persists in a few isolated populations in northern Vietnam and northern and central Lao PDR (Duckworth 2008; Rawson et al. 2011). The populations in Lao PDR are understudied, and the interface with *Nomascus siki* (southern white‐cheeked) has been ambiguous (Hallam et al. 2016; Thinh, Rawson, et al. 2010), although recent research has revealed significant overlap between the two (Coudrat et al. 2025). One of the most critical populations of *N. leucogenys* , and one less likely to be overlapping with *N. siki* , is that in Nam Et–Phou Louey National Park (hereon NEPL) in the northeast of the country (Duckworth 2008). Like their ambiguous global distribution, their distribution within NEPL has been understudied, and the drivers of this distribution are poorly understood. If this species is to persist, it is imperative that conservation efforts at protected areas like NEPL have a better understanding of the species' distribution and the drivers of that distribution.

Even outside Lao PDR, due to gibbons' cryptic behavior and preference for high canopy, traditional transect surveys can be impractical for this group of primates, especially where they exist at low density over large, difficult to access areas (Brockelman and Ali 1987; Nijman and Menken 2005). One of the most common methods for surveying gibbons has become auditory sampling from early morning listening posts, taking advantage of gibbons' early morning song bouts (Brockelman and Ali 1987; O'Brien et al. 2004). Most of these surveys have sought density estimates by employing the triangulation of compass bearings and call timings from multiple listening posts to estimate the number of groups within a given area (Brockelman and Ali 1987; Phoonjampa and Brockelman 2008; Fan et al. 2011; Gilhooly et al. 2015). Because auditory detection rates vary with distance, compass bearings can be significantly inaccurate, and calling rates vary; these methods have required frequent updates to improve accuracy, and different methods of analysis can produce significantly different density estimates (Ray et al. 2015; Vu et al. 2018; Brockelman et al. 2020; Hankinson et al. 2021; Nurvianto et al. 2022).

Using multiple listening posts for triangulation also requires significant resources and reduces the amount of area that can be sampled on a given budget (Cheyne et al. 2016). Using singular listening posts to record simple presence/non‐detection can avoid many of the struggles experienced with density calculation and result in more sampling afforded on a given budget. In established methods of gibbon auditory sampling, listening posts are visited on multiple consecutive mornings to improve the likelihood of detecting all groups in the area, as gibbons do not sing every morning (Brockelman and Ali 1987; O'Brien et al. 2004; Yanuar et al. 2019). Similarly, in occupancy estimation, survey sites are sampled multiple times to estimate the probability of detection (MacKenzie et al. 2018; Long et al. 2011; Kumara et al. 2014).

In 2014, a localized density survey was undertaken for the NEPL *N. leucogenys* population revealing a population at low density (0.4 groups/km^2^) but of global significance (57 groups identified) (Syxaiyakhamthor et al. 2020). The survey revealed a heterogeneous distribution of the species, with relatively high density in some areas of the survey's coverage but with a complete absence in others, despite the apparent suitability of the habitat. The survey failed to reveal correlations with any natural or anthropogenic variables that were satisfying to the authors. Meanwhile, reports from NEPL ranger teams since 2014 revealed the population to be widespread across the park, persisting over much more area than the 2014 density survey had covered.

To estimate the relative rates of occupancy of *N. leucogenys* across NEPL and assess potential predicting variables of this distribution, we conducted presence/non‐detection sampling of sites across the potentially suitable habitat of the entire park. Resulting data were used in a single‐season, single species occupancy modeling framework (MacKenzie et al. 2018) to predict the probability of the propagation of sound of *N. leucogenys'* calls in these sites, in relation to several natural and anthropogenic variables (Gray et al. 2010; Olea and Mateo‐Tomás 2011; Campbell et al. 2019). Propagation of the sound of their calls was used as a proxy for relative rates of occupancy to estimate the effect of these variables on the distribution of the species.

As mentioned, the previous NEPL gibbon density survey (Syxaiyakhamthor et al. 2020) found no satisfying correlations to help explain distribution. We speculated that this was because the primary driver of current distribution was an open war and later insurgency, which involved widespread use of heavy explosives in the landscape and relocation of human communities, lasting from the mid‐1960s through the 1990s. Unfortunately, we had no reliable variable to reflect the distribution of this conflict and human relocation, as old village locations and war artifacts get rapidly obscured by human collection and vegetative growth. Therefore, we could not test this speculation as a hypothesis and were left with the option to test if those earlier results would be replicated when using occupancy modeling of a larger sample size over a less confined survey area. If the results were replicated, it would support our speculation, but if they were not replicated, it might reveal other ongoing drivers of distribution.

## 2 Methods

### 2.1 Study Site

Nam Et—Phou Louey National Park is a conservation priority for Lao PDR, serving as habitat for many of the nation's top priority species and as a model of protected area management (Nam Et—Phou Louey National Park Management Plan). It covers 5070 km^2^ in the north of Lao PDR; divided into two zones: a 3000 km^2^ totally protected zone where access and harvest of all plants and animals is legally restricted, and a 2070 km^2^ controlled use zone which has been designated for sustainable use by the park's human communities, as outlined by the country's 2023 Protected Area Decree. The landscape is a mountainous mosaic of forests, bamboo, and grasslands shaped by a long history of human settlement and shifting cultivation. There are 91 villages in and bordering the park with a combined population of 42,600 people made up of 8 different ethnic groups, primarily Khamu, Hmong, and Lao (Johnson 2012). The park has been habitat to a wide array of large fauna, many endangered, including six species of cat, eight species of non‐human primate, two species of bear, Asian elephants *Elephas maximus*, and over 300 species of birds (Johnson 2012; Davidson 1998; Johnson et al. 2006). While tiger *Panthera tigris* and leopard *Panthera pardus* have been extirpated from the landscape, the park remains vital to a robust population of mainland clouded leopard *Pardofelis nebulosa* and dhole *Cuon alpinus* (Rasphone et al. 2019). There is one species of gibbon in the park, *Nomascus leucogenys* (Duckworth 2008; Coudrat et al. 2025; Syxaiyakhamthor et al. 2020; Thinh, Mootnick, et al. 2010).

### 2.2 Survey Design

**FIGURE 2:**
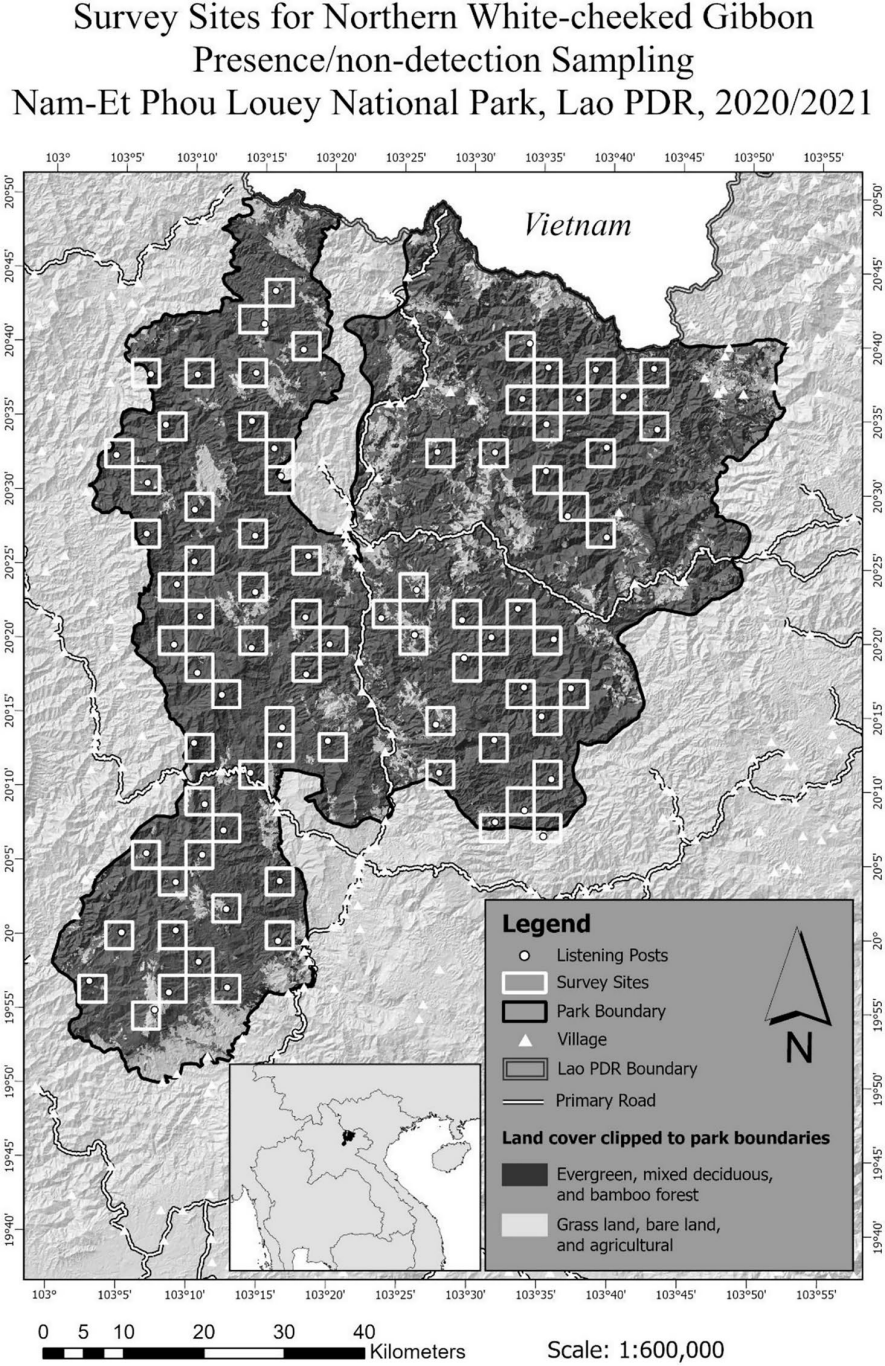
Survey design with 80 semi‐randomly selected sampling sites (each 3.33 × 3.33 km) and 80 pre‐determined listening posts—chosen subjectively to maximize chance of detection within the site and minimize detection from outside.

In this study, survey grid cell size (survey site) was 11.09 km^2^ (3.33 km × 3.33 km). This size was chosen in order to maximize the likelihood that all singing groups inside the site would be detected by a centrally placed, high elevation, listening post, and that there would be a reduced risk of detecting the calls of groups in adjacent sites (non‐independence); detection probability typically dropping off at > 700 m and detection being rare beyond 1.5 km (Brockelman and Ali 1987; Vu et al. 2018). The entirety of NEPL was masked with a grid of 11.09 km^2^ cells and every cell with greater than 50% non‐forest cover (grassland, bare land, and fallow agricultural land) was removed, as determined by a 2011 satellite analysis (Moore et al. 2011). The result was 293 cells (3249 km^2^) determined as potential *N. leucogenys* habitat in NEPL. From these, 60 cells were randomly selected using Hawth's Tools (ArcGIS 9). An additional 20 cells were then selected by the researchers to maximize the breadth of coverage (filling in empty gaps) while avoiding adjacent cells. The result was a semi‐random selection of eighty 11.09 km^2^ sample sites, 887 km^2^ of the 3249 km^2^ of NEPL's potential habitat—about 27% (Figure 2). In each site (cell) a listening post was pre‐determined, subjectively, by the researchers using 1:50,000 topographic maps with 20‐m contours, as a combination of the highest and most central location, where it was expected to have the greatest chance of detecting calls originating from anywhere inside the site.

### 2.3 Data Collection

Each listening post was visited for 4 consecutive mornings, regardless of weather or any other circumstances, from 05:30 (sometimes earlier but never later) to 1 h after the end of the last gibbon call or 10:00 if no calls were heard (Brockelman and Ali 1987; Coudrat et al. 2015). Teams were comprised of 4 surveyors, and listening posts were always stationed by two or more surveyors. The team, on reaching the predetermined listening post on the first morning, was allowed to shift the location of the post by as much as 200 m to achieve what they believed was the closest to the ideal location for detection. The updated point was marked with GPS and used for all 4 mornings. In addition to detection/non‐detection, surveyors recorded estimated distances and compass bearings to call origins, timing of male calls and female *great calls*, numeric weather values from Kestrel weather meters, and categorical values for rain, fog, wind, and clouds. Two adjacent sites were surveyed in late October 2020, as part of a supervised practical training for the surveyors. The other 78 sites were all surveyed between 30 November 2020 and 17 March 2021, by the 16 trained surveyors in 4 individual teams. This time period was selected as it has been demonstrated that gibbons in the area vocalize more frequently in the cool dry season (Coudrat et al. 2015).

In our models of occupancy and detection probability, we integrated a suite of site‐specific covariates, encompassing both natural and anthropogenic factors. Using ArcGIS version 10.6, we computed the focal mean value for each 11.09 km^2^ site, including distance to the nearest road (ROAD), distance to the nearest village (VILLAGE), and distance to the nearest canopy‐breaking river (RIVER). Leveraging ArcGIS alongside a digital elevation model, we derived the focal mean elevation (ELEVATION) and Topographic Roughness Index (TRI) for each site. Additionally, based on the 2011 landcover analysis of NEPL (Moore et al. 2011) within ArcGIS, we quantified the percent cover of bamboo forest (BAMBOO), the combined extent of evergreen and mixed deciduous forest (FOREST), and the proportion of non‐forested areas, including bare land, fallow fields, and grassland (BARE). We also utilized NASA's Fire Information for Resource Management System (FIRMS, accessible at https://firms.modaps.eosdis.nasa.gov/) to tally heat signatures detected by MODIS satellites Terra (MOD14) and Aqua (MYD14) within each site from January 1, 2017, to December 31, 2022 (FIRE). Weather variables included the average temperature across four mornings (TEMP) and the average scores for cloud cover, fog, and rain (each scored from 0 to 2 daily) over the same period (CLOUD, FOG, RAIN). Finally, we constructed a human use index (HUI) for each site using patrol observational data from NEPL ranger teams, sourced from SMART (Spatial Management And Reporting Tool, https://smartconservationtools.org/). The HUI was calculated as the sum of ranger encounters with signs of logging, human camps, gunshots, cable snares, and direct human observation, divided by kilometers patrolled, with all data collected within the survey sites between January 1, 2017, and December 31, 2022. These encounter categories were selected to comprehensively capture every recorded indicator of human activity in the region.

### 2.4 Data Analysis

In our study, we employed the robust methodology for occupancy modeling outlined by MacKenzie et al. (2018) to assess the acoustic dynamics of gibbon calls within a designated site. Utilizing maximum likelihood detection and occupancy models available in the R package *unmarked* (Fiske and Chandler 2011), we estimated the probability of gibbon call sound propagation within the site (denoted as Ѱ) or the probability of site occupancy, and the probability of observers detecting these vocalizations (denoted as *p*). This approach rests on three fundamental assumptions: first, that site occupancy remains stable throughout the season, with no extinction or colonization events (closure); second, that the occupancy of one site operates independently of others (independence); and third, that all detections are true, with no false positives (MacKenzie et al. 2018). These principles underpin our analysis, ensuring a rigorous evaluation of gibbon vocal behavior in their natural habitat.

To ensure the integrity of our study, we carefully addressed potential violations of the three core assumptions underlying our methodology. We minimized breaches of the closure assumption, which requires stability of site occupancy, by limiting each site's sampling season to a concise 4‐day period, reducing the likelihood of extinction or colonization events. Additionally, gibbons live in relatively stable home ranges (compared to other mammals of their size), further minimizing this risk. To uphold the independence assumption, which posits that occupancy at one site does not influence another, we strategically minimized the use of adjacent sites. Where adjacency was unavoidable, we maintained a minimum distance of 2 km between posts, exceeding 3 km for all but one pair, and further mitigated this concern by modeling the occupancy of sound propagation rather than the gibbons themselves, rendering site independence less relevant. Finally, we tackled the risk of false detections by implementing rigorous surveyor training, including exposure to *N. leucogenys* recordings and hands‐on practice with authentic forest calls, while ensuring that each morning's observations at every post involved at least two surveyors, with the lead researcher accompanying each team for at least three sites each to reinforce accuracy and consistency.

We evaluated all identified covariates for their potential influence on detection probability (*p*), with the three weather variables—cloud cover (CLOUD), fog (FOG), and temperature (TEMP) specifically included due to their significant correlation with gibbon calling rates, as demonstrated in a separate analysis of singing behavior (White et al. 2024). All covariates except these weather variables were also considered potential drivers of occupancy (Ѱ). To refine our covariate set, we reduced the initial list to eight (*n*/10) by eliminating those with high Variance Inflation Factor (VIF) scores or strong correlation coefficients. In cases where covariate pairs exhibited multicollinearity and comparable VIF values, we employed Likelihood Ratio Tests on simple models of the covariate in question against *p* or Ѱ, retaining the covariate with the greater likelihood of influencing one or both parameters while discarding the other.

Detection histories from the 80 sites were then integrated into maximum likelihood models using the R package *unmarked*, with all covariates standardized before inclusion. For seven sites where the human use index (HUI) was missing due to ranger patrolling efforts covering less than 10 km, we imputed the median HUI value. For comparison, we also ran this analysis on an alternative dataset where all sites with missing HUI values were removed entirely.

A two‐step modeling approach was adopted, where first, detection probability (*p*) was modeled by varying all possible combinations of detection covariates with occupancy held constant. Second, occupancy (Ѱ) was modeled by varying all possible combinations of occupancy covariates with detection (*p*) fixed to the previously best identified detection model.

Models were ranked using Akaike information criterion—corrected (AICc). AICc is a value based on maximum likelihood estimation and the minimization of parameters (parsimony) (Olea and Mateo‐Tomás 2011; Campbell et al. 2019). ΔAICc is the measure of the difference between the best model and the subsequent model; models with ΔAICc < 2 are considered to have greater support (MacKenzie et al. 2018). Finally, we evaluated the goodness‐of‐fit for selected models (those within 2 ΔAICc of the top model) using 1000 bootstrap iterations.

## 3 Results

We recorded 154 *N. leucogenys* song bouts, 137 of them duets and 17 male solos with no female solos over the 320 survey days with a mean of 0.48 bouts per day. These song bouts were detected at 34 of 80 sites, giving a naïve occupancy of 0.43.

**FIGURE 3:**
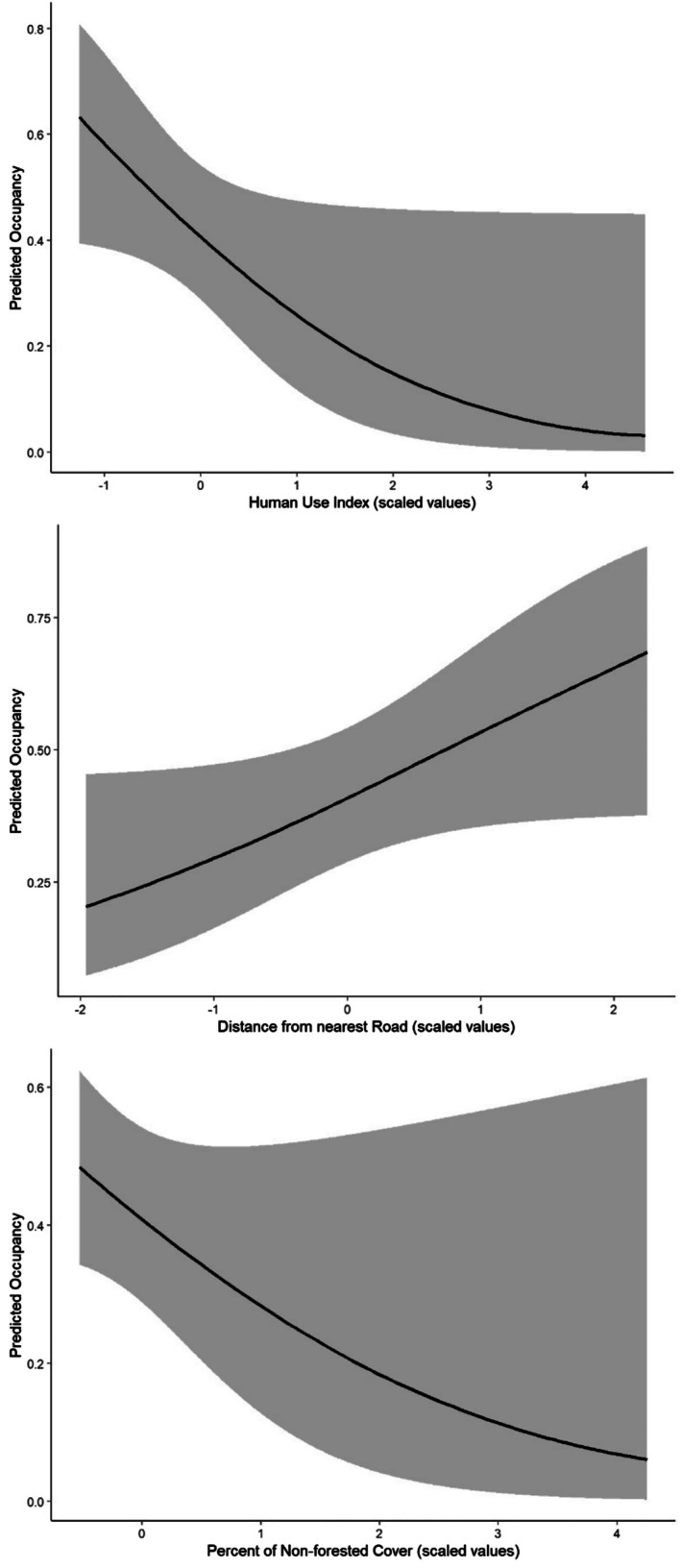
Predictive trends by the three covariates for occupancy from the top‐ranking model.

From the top‐ranking model, occupancy probability of the sound propagation of gibbon calls displayed a strong and significant negative correlation with the human use index (*β* = −0.69, *p* = 0.05), a weaker and nonsignificant positive correlation with distance from the nearest road (*β* = 0.5, *p* = 0.06), and a negative nonsignificant correlation with the percent of non‐forest cover in the site (*β* = −0.56, *p* = 0.12). Detection probability had a strong but nonsignificant positive correlation with cloud cover (*β* = 0.68, *p* = 0.07), a negative and significant correlation with percent of bamboo forest cover (*β* = −0.48, *p* = 0.05), and a negative and nonsignificant correlation with the topographic roughness index (*β* = −0.47, *p* = 0.08). 1000 bootstraps of this model resulted in model convergence, suggesting a good fit. The predicted occupancy rate from this model was 0.44, or 44% occupancy of the surveyed area. These results can be seen in Tables 1 and 2 and Figure 3, where the probability of gibbon occupancy (proxied by occupation of sound propagation) in NEPL is greater with less human use, greater distance from roads, and less forest cover interruption. The alternative running of the analysis in which sites that had missing HUI values were removed, rather than median HUI values used, produced insignificant differences in results.

**TABLE 1:** Top ranking models for occupancy and detection.

Model	*K*	AICc	∆AICc	AICc Wt	Cum. Wt	LL
Ѱ (HUI + ROAD + BARE) *p* (CLOUD + BAMBOO + TRI)	8	260.46	0.00	0.26	0.26	−121.22
Ѱ (HUI + ROAD) *p* (CLOUD + BAMBOO + TRI)	7	261.30	0.84	0.17	0.44	−122.87
Ѱ (HUI + BARE) *p* (CLOUD + BAMBOO + TRI)	7	261.84	1.37	0.13	0.57	−123.14
Ѱ (BARE + ROAD) *p* (CLOUD + BAMBOO + TRI)	7	263.01	2.55	0.07	0.64	−123.73
Ѱ (ROAD) *p* (CLOUD + BAMBOO + TRI)	6	263.17	2.70	0.07	0.71	−125.01
Ѱ (HUI) *p* (CLOUD + BAMBOO + TRI)	6	264.56	4.10	0.03	0.74	−125.70
Ѱ (ROAD + TRI) *p* (CLOUD + BAMBOO + TRI)	7	264.63	4.17	0.03	0.78	−124.54
Ѱ (ROAD + RIVER) *p* (CLOUD + BAMBOO + TRI)	7	264.77	4.31	0.03	0.81	−124.61
Ѱ (HUI + BAMBOO) *p* (CLOUD + BAMBOO + TRI)	7	264.92	4.46	0.03	0.83	−124.68
Ѱ (BARE) *p* (CLOUD + BAMBOO + TRI)	6	265.18	4.71	0.02	0.86	−126.01

**TABLE 2:** Top ranking model for occupancy and detection (∆AICc = 0).

Ѱ (HUI + ROAD + BARE) *p* (CLOUD + BAMBOO + TRI)				
	*β*	SE	*z*	*p*
Occupancy (logit scale)				
Intercept	−0.368	0.273	−1.35	0.1782
HUI	−0.692	0.355	−1.95	0.0514
ROAD	0.504	0.266	1.89	0.0583
BARE	−0.562	0.366	−1.53	0.1251
Detection (logit scale)				
Intercept	0.963	0.241	3.99	0.00007
CLOUD	0.675	0.366	1.84	0.0653
BAMBOO	−0.484	0.247	−1.96	0.0497
TRI	−0.466	0.262	−1.78	0.0759
AIC: 258.4364				
Number of sites: 80				
Optimum convergence code: 0				
Optimum iterations: 31				
Bootstrap iterations: 1000				

## 4 Discussion

This study demonstrated the efficacy of combining acoustic and occupancy survey techniques to suggest factors influencing the distribution of the northern white‐cheeked gibbon. This integrated approach successfully addressed challenges inherent in surveying arboreal species with distinctive terrestrial vocalizations that (1) exist at very low and heterogeneous population densities, (2) are dispersed unpredictably across extensive, topographically complex landscapes, and (3) exhibit extreme wariness toward human presence.

We believe a similar survey design and analysis can be applied for other vocally distinctive species and clades, and does not need to be limited to use with human detectors, but can also be employed with autonomous auditory recorders. In Lao PDR, just as an example, species of similar critical conservation importance that could be surveyed in this way include the Vietnamese crested argus ( *Rheinardia ocellata* ), coral‐billed ground cuckoo ( *Carpococcyx renauldi* ), and Siamese crocodile ( *Crocodylus siamensis* ). An important consideration for these surveys is choosing an appropriately sized sampling site, which is based on an accurate range of distances from recorder for probable detection. This will depend on the species being surveyed and the detection device being used. For gibbons with human detectors, we had these ranges already tested from prior studies (Brockelman and Ali 1987; Vu et al. 2018), but for other species and autonomous recording devices, these should be tested first to avoid underdetection or violation of the independence assumption (spatial autocorrelation).

Our methodology effectively identified factors likely to be of great importance to the conservation of this Critically Endangered species, suggesting key drivers of their sparse and fragmented distribution. Furthermore, this approach yielded novel insights regarding detection variables, particularly those affecting sound propagation and attenuation in the forest environment—findings that should enhance the design and implementation of future bioacoustic surveys for gibbons and other vocally distinctive species.

Findings from this study did not replicate the results of the earlier NEPL gibbon survey, which found no variables strongly suggestive of driving distribution, and therefore, our findings did not support our speculation that distribution was largely the result of past human conflict and community relocation. Instead, we see evidence that fragmentation of habitat and human presence in that habitat are important drivers of the species' distribution. It is still likely that past historic events had an impact on the species' distribution, but we now have evidence to suggest that there are ongoing factors still affecting the continued occupancy of the species in the forests of the park.

The three ranking covariates of detection (*p*) reveal much about the propagation and attenuation of gibbon call sound. The reduced detection experienced in more topographically rugged sites is easy to understand: deep valleys and high ridges create more barriers to sound propagation. The negative correlation of detection with bamboo forest fits with various prior research finding greater attenuation of high‐frequency sound with greater forest structure (Martens 1980; White and Swearingen 2004; Ziemann et al. 2013; Boycott et al. 2019); and with previous comparisons of sound attenuation over different forest types (Sakai et al. 2001), which observed a greater reduction in dB across bamboo forest than across the same distance of non‐bamboo forest.

The increased detection under cloud cover suggests that high altitude atmospheric humidity affects the long‐distance propagation of gibbon call sound and thus its detection rates. This is the second instance that we know of where cloud cover was associated with improved detection of gibbon vocalization. Brockelman and Srikosamatara (1993) found a significant but weak correlation between cloud cover and detection of *Hylobates pileatus* in Thailand. Furthermore, it is consistent with a separate analysis conducted from this survey, where the number of daily detected gibbon song bouts was found to positively correlate with cloud cover and was assumed to be a function of detection (White et al. 2024). The literature on meteorological effects on sound propagation typically finds wind to affect sound propagation more than other meteorological variables (Ingård 1953; Van Renterghem and Botteldooren 2010), but we did not find a significant difference in song bouts detected between days with and without wind (White et al. 2024). More in line with our results concerning cloud cover, Larsson (1997) found atmospheric absorption of sound to relate to relative humidity. While we did not find evidence to suggest relative humidity affects singing behavior or detection, our measurements of relative humidity were made at ground level, under the canopy, and might not reflect higher altitude atmospheric humidity (White et al. 2024). To conclude, we found detection to improve with cloud cover and to worsen with greater topographic complexity and with more bamboo forest.

For the covariates best predicting occupancy (Ѱ), the proportion of the site non‐forested correlating negatively with occupancy is not surprising, as it is well known that gibbons do not normally occupy grasslands or unforested agricultural lands. The covariates of Human Use Index and distance from the nearest road being the most significant of all covariates modeled are more noteworthy for a few reasons. First, this is evidence that human presence in the habitat is a greater driver of distribution than natural variables, even more significant than the amount of unforested land in a site. Second, the correlation of distance from road supports the findings of other published (Hallam et al. 2016) and unpublished WCS gibbon occupancy surveys in Lao PDR, which have found distance from road to be the best predictor of gibbon occupancy. Finally, this model adds credence to the assumption that distance from road serves as a proxy of human presence, although we did not find multicollinearity between these two covariates.

**FIGURE 4:**
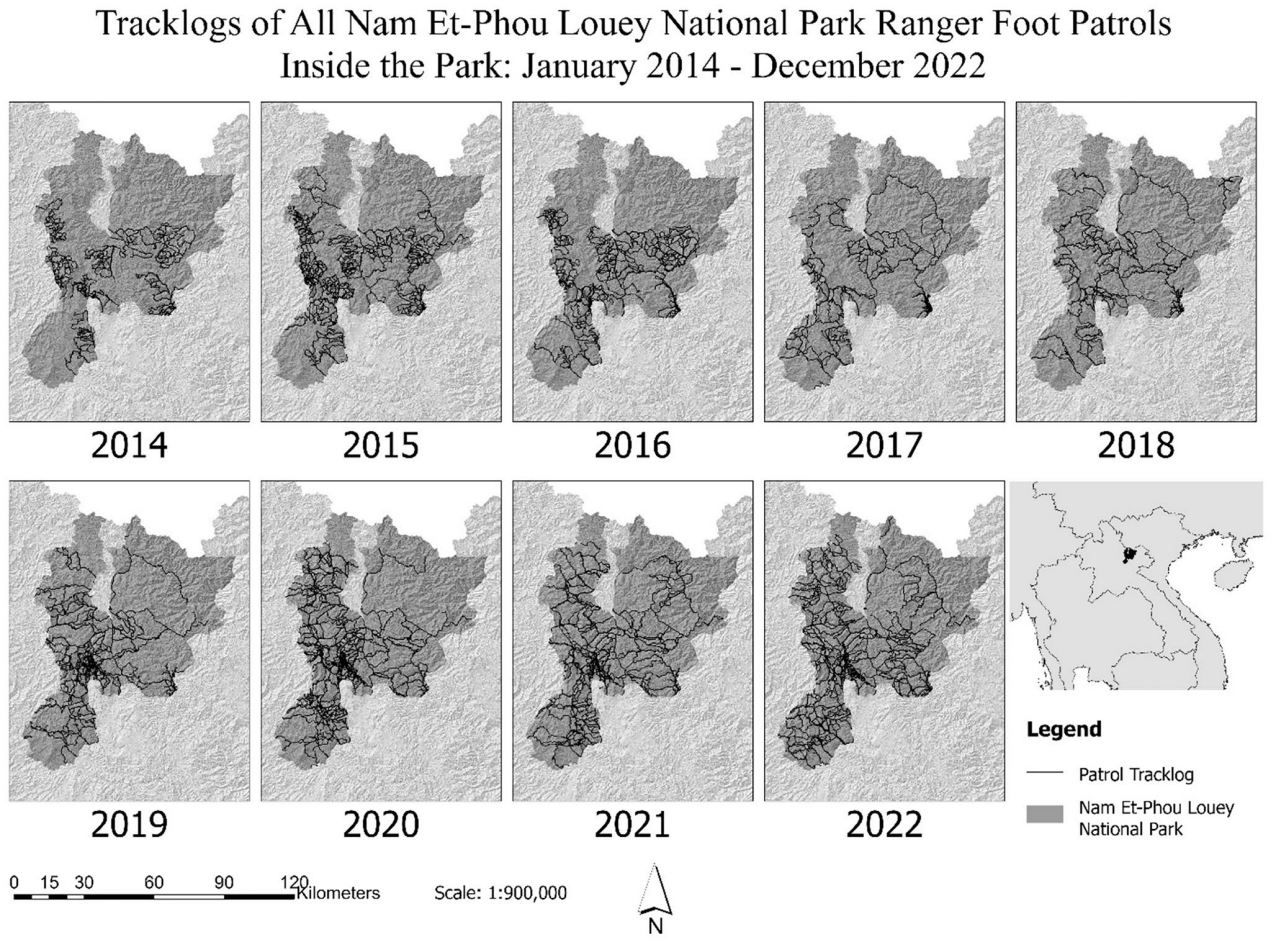
Patrol tracklogs of NEPL ranger teams 2014–2022. Prior to 2014, patrols used waypoints and did not record tracklogs. Each year shows the track logs of all forest ranger patrols between the dates January 1 and December 31. All but one sub‐station was closed in the latter half of 2016 and teams were reduced in number and changed to a mobile strategy of being deployed to patrols by car; in 2019 funding allowed teams to increase and maintain a combination of sub‐station and mobile strategies.

This is not the first time NEPL ranger data have been used as a covariate in modeling gibbon distribution in the park. Analysis of the 2014 NEPL gibbon density survey (Syxaiyakhamthor et al. 2020) also used NEPL ranger patrol results collected at the time of analysis (2017) as a covariate but found no correlation with gibbon density. The difference between those results and ours is due to (1) the greater breadth of coverage of the 2021 occupancy survey compared to the 2014 density survey and (2) an overhaul in patrol strategy in 2016. The 2014 density survey focused on one region in the north‐east of the park, while the 2021 occupancy survey had semi‐random coverage across the entire park. The SMART patrol data available to these researchers in 2017 derived almost entirely from sub‐station‐based ranger teams. The sub‐station rangers were able to patrol intensively in the areas immediately outside the sub‐stations, but they were not reaching large portions of the park; therefore, their patrol data was not representative of the whole park (Figure 4). Additionally, the 2014 density survey measured rate of encounter by number of encounters in 1 km^2^ grid cells/number of times rangers entered the grid cell. This was because ranger patrols were recorded by waypoints as opposed to track logs prior to 2014. In 2016, all but one of the sub‐stations were shut down, and patrol operations moved to mobile operations in which teams were delivered and received by car and patrolled based on strategic need rather than proximity to a sub‐station. Large areas of the park were no longer unpatrolled (Figure 4) and people in the park had greater difficulty predicting and avoiding patrols (personal observation). Six years of broader patrol coverage (2017–2022), with an improved ability to encounter people; a more accurate method of measuring patrol effort (track logs as opposed to waypoints); and a broader coverage of the gibbon survey provides more representative data for rates of human use and gibbon occurrence.

Other examples of wild mammal density or occupancy declining in relation to proximity of human use are common globally (Kumara et al. 2014; Feng et al. 2021; Reece et al. 2023; Regmi et al. 2023), including for gibbons (Phoonjampa and Brockelman 2008; Ray et al. 2015; Zhang et al. 2020; Phung et al. 2023). While occupancy modeling of *N. gabriellae* in Cambodia (Gray et al. 2010) did not find occupancy to relate to distance from roads (assumed as a proxy of human usage), Hallam et al. (2016) did find distance from roads as the best predictor for occupancy of *N. leucogenys/siki* in central Lao PDR. Recent occupancy surveys of gibbons in other parts of the country have had similar results; in the absence of long‐term detailed human presence data, distance from roads repeatedly serves as the best predictor of gibbon occupancy in northern and central Lao PDR (unpublished WCS occupancy surveys). While in countries such as India and Thailand gibbon occurrence can readily be observed adjacent to roads inside protected areas, these countries have much greater natural resource protection efforts and control of road use in protected areas, making hunting along these roads much more difficult than in Lao PDR (personal observation). These findings therefore provide evidence that one of the most important actions to take for the continued viability of the six species of gibbon in Lao PDR (PDR Lao 2011) (in the absence of an unlikely enormous and immediate improvement of protected area management) is to prevent the construction of roads into these remaining habitats.

In mainland Southeast Asia, and in particular Lao PDR, rampant hunting has been demonstrated to be a greater driver of defaunation than habitat degradation, resulting in the ‘empty forest syndrome,’ which has become emblematic of Lao protected areas (Gray et al. 2017; Rasphone et al. 2019, 2021; Hughes 2017; Tilker et al. 2019; Groenenberg et al. 2023). Over the course of a decade in NEPL, tiger, leopard, and gaur *Bos gaurus* were all extirpated from the park's forests through the employment of snares and foothold traps, firearms, hunting dogs, and explosives (Rasphone et al. 2019; Johnson et al. 2006). While it has been documented that hunters of some communities in mainland Southeast Asia avoid the killing of gibbons due to social taboos (Duckworth 2008) these taboos are not widely expressed around NEPL and hunting in the park, in general, is unselective and opportunistic (personal observation and communication). Furthermore, advertisements for live *Nomascus* gibbons on social media accounts in Lao PDR evidence that there is harvest of the species occurring (Facebook ‘ກູ່ມສາຍລ່າແລະຮັບຊື້ສັດປ່າ’, 2023).

There is considerable criticism of the ‘fortress park’ model of protected area management (Bell 1988; Knight 2000; Kabra 2019; Murdock 2021). There are ongoing global and local pressures for park administrators and conservationists to abandon this model. In NEPL, this pressure is typified by the encouragement to allow local communities greater access to the totally protected zone of the park for cattle grazing and collection of non‐timber forest products (World Bank report by Foppes and Sayalath, 2022; personal communication). There are sound hypothetical arguments for this relaxing of restrictions from a social‐political perspective; increasing the benefits that communities receive from natural resources may encourage them to better protect them for long‐term sustainability. However, these arguments are likely only to apply when an adequate system of law enforcement is present to prevent this relaxation for local communities from being taken advantage of by others, and when use can be effectively monitored and regulations effectively enforced. In the current state of natural resource use and management in Lao PDR, our results reveal that, at least for the viability of *Nomascus leucogenys* , improving human access to these habitats at this time will hinder, not help, their chances of survival.

## Author Contributions

**Jay White:** conceptualization (equal), data curation (equal), formal analysis (lead), funding acquisition (equal), investigation (equal), methodology (equal), project administration (equal), writing – original draft (lead), writing – review and editing (equal). **Akchousanh Rasphone:** conceptualization (equal), data curation (equal), formal analysis (equal), investigation (equal), methodology (equal), writing – review and editing (equal). **Khamkeo Syxaiyakhamthor:** conceptualization (supporting), data curation (supporting), formal analysis (supporting), funding acquisition (equal). **Anong Thorya:** data curation (equal), formal analysis (supporting), investigation (supporting), resources (supporting).

## Ethics Statement

This research abided by the Ecology and Evolution guidelines on ethical standards and was carried out in accordance with the laws of Lao PDR. The lead author from Wildlife Conservation Society (WCS) Lao PDR falls under the Memorandum of Understanding (signed January 26, 2023) between the Lao Ministry of Agriculture and Forestry and WCS Lao PDR to serve as a technical adviser for Nam Et–Phou Louey, and this research falls under his responsibilities to aid the park management unit.

## Conflicts of Interest

The authors declare no conflicts of interest.

## Supporting information


**Data S1:** ece372957‐sup‐0001‐supinfo.zip.

## Data Availability

The data that support the findings of this study are available on request from the corresponding author. All the required data are uploaded as Supporting Information.
